# Lithium placental passage at delivery and neonatal outcomes: A retrospective observational study

**DOI:** 10.1192/j.eurpsy.2021.540

**Published:** 2021-08-13

**Authors:** M.L. Imaz, M. Torra, D. Soy, K. Langorh, L. Garcia-Esteve, R. Martin-Santos

**Affiliations:** 1 Unit Of Perinatal Mental Health Clinic-bcn, Department Of Psychiatry And Psychology, Institut Of Neuroscience, Institut D’investigacions Biomèdiques August Pi I Sunyer (idibaps), And Department Of Medicine, University Of Barcelona (ub), Hospital Clinic Barcelona, Barcelona, Spain; 2 Pharmacology And Toxicology Laboratory, Biochemistry And Molecular Genetics Service, Biomedical Diagnostic Center, Idibaps, Hospital Clinic Barcelona, Barcelona, Spain; 3 Division Of Medicines, Pharmacy Service, Idibaps, Hospital Clinic Barcelona, Barcelona, Spain; 4 Grass Research Group In Survival Analysis. Department Of Statistic And Operations Research., Universitat Politècnica de Catalunya, Barcelona, Spain; 5 Unit Of Perinatal Mental Health, Department Of Psychiatry And Psychology, Idibaps, Hospital Clinic Barcelona, Barcelona, Spain; 6 Psychiatry And Psychology Department, Cibersam, Idibaps, Ub, Hospital Clinic, Barcelona, Spain

**Keywords:** Neonate, lithium, Placental transfer, Delivery

## Abstract

**Introduction:**

Lithium is an effective mood stabilizer and is widely used as a first-line treatment for bipolar disorder in the perinatal period. Several guidelines have provided clinical advice on dosing strategy (dose reduction versus stop lithium) in the peripartum period to minimize the risk of neonatal complications. An association has been observed between high neonatal lithium concentrations (> 0.64 mEq/L) and lower 1-min Apgar scores, longer hospital stays, and central nervous system and neuromuscular complications.

**Objectives:**

To quantify the rate of lithium placental passage at delivery. To assess any association between plasma concentration of lithium at delivery and neonatal outcome.

**Methods:**

In this retrospective observational cohort study, we included women treated with llithium at least in late pregnancy. Maternal (MB) and umbilical cord (UC) lithium blood level measurement were collected at delivery. Lithium serum concentrations were determined by means of an AVL 9180 electrolyte analyzer. The limit of quantification (LoQ) was 0.20 mEq/L and detection limit was 0.10 mEq/L. From the medical records, we extracted information on neonatal outcomes (preterm birth, birth weight, Apgar scores, pH-values, and admision to NICU) and complications categoriced by organ system: respiratory, circulatory, hematological, gastro-intestinal, metabolic, neurological, and immune system (infections).

**Results:**

Umbilical cord and maternal lithium blood levels were strongly correlated: mean (SD) range UC/MR ratio 1.15 (0.24). Umbilical cord lithium levels ranged between 0.20 to 1.42 mEq/L. We observed no associations between umbilical cord lithium blood levels at delivery and neonatal outcomes.

**Conclusions:**

In our study, newborns tolerated well a wide range of lithemias, between 0.20 and 1.42 mEq/L.

